# Video monitoring of brown planthopper predation in rice shows flaws of sentinel methods

**DOI:** 10.1038/srep42210

**Published:** 2017-02-17

**Authors:** Yi Zou, Joop de Kraker, Felix J. J. A. Bianchi, Mario D. van Telgen, Haijun Xiao, Wopke van der Werf

**Affiliations:** 1 Centre for Crop Systems Analysis, Wageningen University, Wageningen, The Netherlands; 2 Department of Science, Open University, Heerlen, The Netherlands; 3 Farming Systems Ecology, Wageningen University, Wageningen, The Netherlands; 4 Institute of Entomology, Jiangxi Agricultural University, Nanchang, China

## Abstract

Immobilized preys are routinely used in agro-ecological exposure studies to quantify predation of pests under field conditions, but this method has not been validated. Our purpose was to determine the validity of using immobilized adults of the major rice pest *Nilaparvata lugens*, brown plant hopper (BPH), as sentinels. We used direct observation by video recording to determine the causal agents of removal of field exposed BPH sentinels with two experiments: 1) we recorded removal events of dead, immobilized BPH; and 2) we compared removal of (i) dead, immobilized BPH, (ii) live, immobilized BPH, and (iii) live, mobile BPH. Long-horned grasshoppers were responsible for most removals of dead, immobilized BPH, in both experiments. Predatory ground beetles removed most of the live, immobilized BPH, whereas frogs were the major predators of live, mobile BPH. Overall, we showed that removal of immobilized sentinel prey is not representative for predation of live, mobile prey, stressing the need for a critical assessment of commonly used sentinel methods. In addition, we found that frogs played the major role in predation of BPH in rice. As current strategies to enhance biocontrol of planthoppers in rice focus on arthropod natural enemies, this finding could have major implications.

Natural pest suppression by predators is an important ecosystem service in agroecosystems, but quantification of predation is challenging. A common method to measure predation is the placement of immobile prey in the field (referred to as “sentinel” or “prey enrichment”) and quantifying the prey removal rate by comparing the number of prey before and after a known exposure period^1^,^2^,^3^. Sentinel methods are widely used because they are quick, cheap and easy to carry out when compared to alternative methods, such as predator exclusion.

A wide variety of sentinel prey types have been used, including insect eggs^4^,^5^,^6^,^7^,^8^, pupae^9^,^10^,^11^, immobilized larvae or adults^12^,^13^,^14^ and artificial prey made from plasticine^15^,^16^,^17^,^18^,^19^. While some insect stages are sedentary by nature (eggs, pupae), mobile insect stages (nymphs, larvae and adults) may be immobilized to facilitate placement and retrieval in the field, for instance by fixing them on cards. Here we focus on the assessment of predation using immobilized insect stages, a method that has been widely used (Table 1).

The use of immobilized prey to estimate predation levels in agroecosystems has some potential biases. First, the probability of a removal event of immobilized prey may be higher than for unmanipulated prey because of their inability to escape or defend themselves. Second, natural enemies may have a preference for either mobile or immobile prey. For example, Brooks, *et al*.^20^ found a higher predation rate of live, mobile than of dead, immobilized prey in a freshwater macroinvertebrate system, while Steward, *et al*.^21^ reported that predatory wasps (Vespidae) preferred pinned to unpinned larvae. Hence, the method of prey manipulation can affect the estimation of predation rates.

An important aspect of determining the validity of a sentinel method is to assess whether the removal agent of immobilized prey is also a predator of the prey in unmanipulated settings. Direct observation can provide firsthand information about the predators involved in pest suppression^22^,^23^,^24^,^25^,^26^, but this method is laborious and difficult at night and under adverse weather conditions. Video recording of exposed prey can resolve these limitations as it allows continuous monitoring for extended periods under a wide range of environmental conditions. Yet, only a very limited number of studies have observed predation of sentinel prey by video recording^11^,^19^,^27^, and we are not aware of studies that tested the validity of immobilized sentinel preys using video recording of predation.

Here we studied the removal of brown planthopper (BPH) *Nilaparvata lugens* Stål (Homoptera: Delphacidae) in rice using video cameras. BPH is one of the major rice pests in Asia^28^. Varietal resistance and insecticides are the main strategies for BPH control in Asia, but outbreaks may still occur as a result of breakdown of varietal resistance to BPH, resistance development in BPH against the insecticides used, and decimation of natural enemy populations by pesticides^29^. Biological control for BPH by natural enemies (predators, parasitoids and pathogens) is critically needed to prevent outbreaks and reduce the side effects of pesticides^30^.

There are over 160 reported species of predators of planthoppers in rice, mostly invertebrates, with spiders (Araneae) being the dominant group^31^. Predators are attributed a major role in natural pest suppression of BPH^28^,^32^,^33^. The impact of predators on BPH has been quantified mainly using predator exclusion and analysis of predator and prey population dynamics^34^. Sentinel methods have been used for BPH eggs^5^, but not for assessment of predation of nymphs or adults.

The objective of our study was to determine the validity of using adult BPH as a sentinel to quantify predation levels in rice. We asked two research questions: (i) what is the identity and relative importance of the agents responsible for removal of dead, immobilized BPH, and (ii) is the removal of immobilized BPH representative of BPH predation under unmanipulated conditions? We carried out two experiments to address these questions. In a first experiment, we recorded removal events of immobilized, dead BPH. In a second experiment, we used a comparative approach with three treatments and recorded agents removing (1) dead, immobilized BPH, (2) live, immobilized BPH, and (3) live, mobile BPH.

## Results

**Figure 1:**
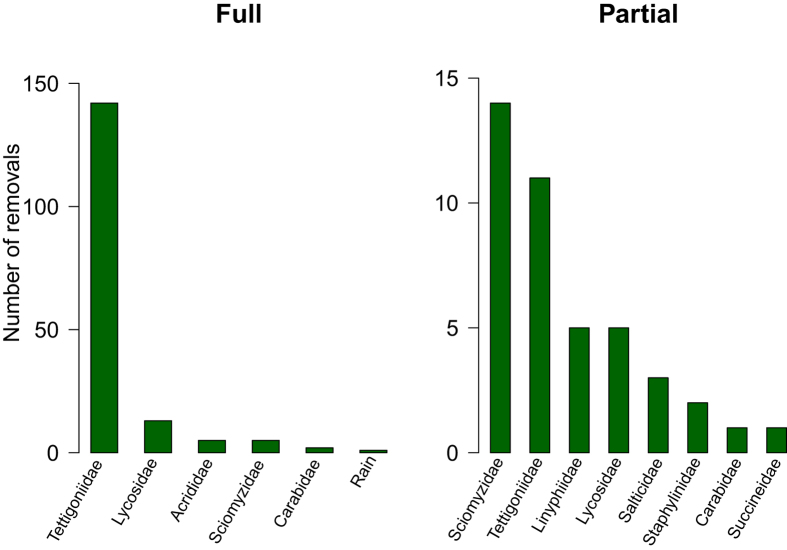
Number of full removals and partial removals of brown plant hopper by different consumer taxa and rain in Experiment 1.

In the first experiment, 168 full removals (the removal of the entire body of a BPH) and 42 partial removals (the removal of only a part of the BPH body) of dead, immobilized BPH were recorded out of 250 BPH exposed (Supplementary material 1). Long-horned grasshoppers (Tettigoniidae: *Conocephalus longipennis*) were responsible for 85% of the full removals, while the contribution of known BPH predators such as wolf spiders (Lycosidae) and ground beetles (Carabidae) accounted for only 9% of the full removals (Fig. 1). Marsh flies (Sciomyzidae: *Sepedon* spp.) and long-horned grasshoppers (Tettigoniidae: *C. longipennis*) were the main agents responsible for partial removal of BPH, with again a minor role for spiders (Linyphiidae, Lycosidae, Salticidae) and predatory beetles (Staphylinidae, Carabidae) (Fig. 1).

In the second experiment, a total of 44 full removals and 28 partial removals of dead immobilized BPH were recorded out of 150 BPH, and 66 full removals and 25 partial removals of live, immobilized BPH, also out of 150. Full removals were significantly more prevalent with live, immobilized BPH than with dead BPH (generalized linear mixed model, z = 2.11, *P* = 0.035), while partial removals were not different between these two treatments (z = −0.42, *P* = 0.68). For the live, mobile BPH treatment, we recorded 93 full removals out of 634 exposed and freely moving BPH, while 493 left the monitored rice stems.

**Figure 2:**
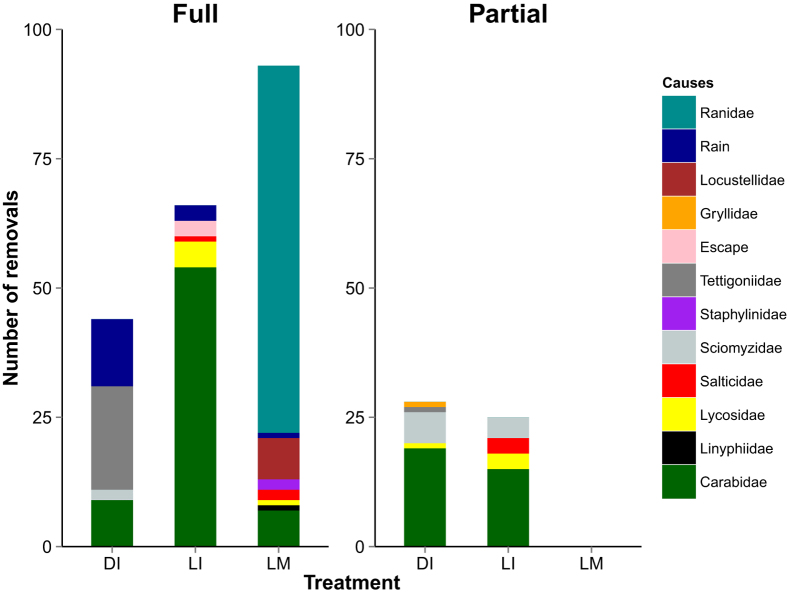
Experiment 2: number of full removals and number of partial removals of brown plant hopper by different causes in three treatments (DI: dead immobilized; LI: live immobilized; LM: live mobile).

There were major differences in the taxa responsible for removal of BPH in the three treatments (dead immobilized BPH, live immobilized BPH, and live mobile BPH) (Fig. 2, Supplementary material 2). Long-horned grasshoppers (*C. longipennis*) removed 20 dead, immobilized BPH (45%), rain caused 30% of the removals, while ground beetles (Carabidae) and marsh flies (Sciomyzidae: *Sepedon* spp.) caused a major part of partial removals, 68% and 21% respectively. The major taxa responsible for removal of live, immobilized BPH were ground beetles (82%) and wolf spiders (Lycosidae, 8%). In addition, BPH escaped occasionally from the sticky tape. The major taxa for partial removals of live, immobilized BPH were again ground beetles (60%) and marsh flies (27%). The major taxa removing live mobile BPH were frogs (Ranidae: *Rana limnocharis*), which removed 70 (75%) BPH (Fig. 2); the warbler bird *Locustella ochotensis* (Locustellidae) was responsible for the removal of 8 live, mobile BPH during two visits (see Supplementary material 3 for video recording of selected predators).

**Figure 3:**
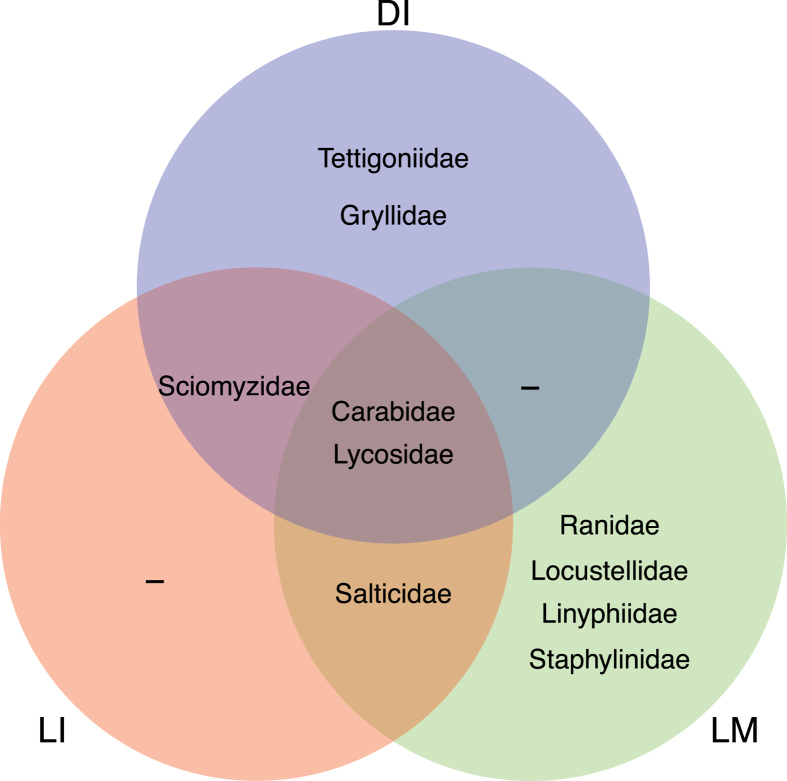
Taxa responsible for removal of brown plant hopper in three treatments (DI: dead immobilized; LI: live immobilized; LM: live mobile) in Experiment 2.

There was thus strikingly little accordance in the taxa mainly responsible for BPH removal in the three treatments. Ground beetles (Carabidae) and wolf spiders (Lycosidae) were the only two taxa that removed BPH in all treatments (Fig. 3). Long-horned grasshoppers (Tettigoniidae) and crickets (Gryllidae) were only observed to remove dead, immobilized BPH, while frogs (Ranidae), warbler birds (Locustellidae), rove beetles (Staphylinidae) and dwarf spiders (Linyphiidae) were only observed to remove live, mobile BPH (Fig. 3).

The success rate (removals/attempts) for ground beetles (Carabidae) was significantly higher in the treatments with immobilized BPH than in case of live, mobile BPH (χ^2^ = 17.8, df = 1, P < 0.001, and χ^2^ = 8.9, *P* = 0.003). No significant differences in success rate were found between the two treatments with immobilized BPH for both ground beetles (Carabidae) and wolf spiders (Lycosidae). Frogs rarely tried to remove dead, immobilized BPH (1 attempt by *Rana limnocharis*) or live, immobilized BPH (3 attempts by *Pelophylax nigromaculatus*), but these attempts were not successful.

During the second experiment, the abundance of arthropods in the study field was assessed with 36 blower-vac samples. Focusing on arthropod predators responsible for removal of live, mobile BPH (treatment 3), ground beetles (Carabidae) had the highest abundance (38 specimens caught), followed by wolf spiders (Lycosidae, 31) and money spiders (Linyphiidae, 30). Low numbers of jumping spiders (Salticidae, 10), and rove beetles (Staphylinidae, 2) were found. There was no significant relationship between the abundance of arthropod predator taxa (as estimated by blower-vac sampling) and the number of live, mobile BPH removed by these taxa (*P* = 0.35).

The results of the second experiment confirm those of the first experiment with respect to the major role of the long-horned grasshopper *C. longinpennis* in full removal of dead, immobilized BPH (Figs 1 and 2). Differences concerned a more prominent role of Carabidae in treatment 1 of experiment 2, and, in particular, a large impact of rain. In the first experiment, rain was responsible for less than 1% of all full removals of dead, immobilized BPH, but in the second experiment rain was responsible for about 30% of losses. The removals happened mainly (92%) during a single intense rain event delivering 21 mm within one hour during the second experiment. In comparison, the most intense daily rainfall during the first experiment was 18.4 mm deposited over a few hours.

## Discussion

Our data demonstrate that (1) the agents responsible for removal of immobilized BPH sentinels differed greatly in identity and relative importance from the taxa found preying on live, mobile BPH, and (2) frogs played a major role in removal of live, mobile BPH.

The taxa we recorded removing live, mobile BPH are all known predators of BPH^31^,^35^,^36^, with the exception of the warbler bird *L. ochotensis*, which represents a first time record. In contrast, the agents recorded fully removing dead, immobilized hoppers were a mix of taxa known as omnivores (Tettigoniidae), scavengers (Sciomyzidae), true predators (Lycosidae, Carabidae), and even herbivores (Acrididae)^37^. The longhorned grasshopper *C. longipennis* (Tettigoniidae), responsible for most removals of dead, immobilized BPH in both experiments, is an opportunistic omnivore known to feed on parts of the rice plant as well as stemborer egg masses^38^,^39^. Records of *C. longipennis* consuming live, mobile nymphs and adults of BPH are based on inclusion tests conducted in the laboratory^40^,^41^. As *C. longipennis* removed only dead, immobilized BPH and not any live BPH, this species should not be considered to play a role in predation of BPH under field conditions.

Other notable differences between the treatments in experiment 2, were the large impact of rain and the significantly higher success rate of carabid predators in full removal of dead, immobilized BPH as compared to live, mobile BPH. Taking the overall ratio of full removals over the total number of BPH exposed as an approximation, the overall removal proportions in live, mobile BPH was highest (93/141, corrected for departures), followed by live, immobilized BPH (66/150) and dead BPH (44/150). The difference in removals and success rate between live, immobilized BPH and dead BPH was only small, which is likely due to the rather short survival time in the field of live immobilized BPH (50% mortality after about 3 hours). We conclude that removal of immobilized BPH (either dead or live) is not representative of predation of live, mobile BPH and exposure of immobilized BPH as sentinels is not a valid method to quantify predation levels in rice fields.

Using direct observation with the explicit aim to validate sentinel methods has not been done before, probably because direct observation is laborious and difficult to carry out. However, with the current availability of affordable, high-quality video cameras with night vision, direct observation has become a feasible and powerful approach to validate and complement sentinel methods.

Our study is the first to use direct observation and video monitoring to study predation of BPH in rice. Remarkably, despite the pest status of BPH and the major role attributed to predators in its natural control, no studies have been published on the identity and relative importance of predators of BPH based on direct observation of predation in the field. This might explain why the prominent role of frogs in BPH predation as found in our study has so far gone unnoticed, attributing the major role to spiders and water bugs^32^,^42^.

Frog species, including *R. limnocharis*, have been previously recognized as potential predators of BPH^35^,^36^. However, their contribution to pest suppression has, so far, not been considered significant^37^. This is surprising considering the abundance of frogs in rice agro-ecosystems and the importance of insects in the diet of frogs^43^. Quantitative studies on the role of frogs in BPH predation are lacking, and major text books on integrated and biocontrol of rice pests^30^,^44^ as well as key publications on assessment of the role of natural enemies in rice pest management^34^,^45^ only focus on arthropods but see^43^. Although our findings on the role of frogs and birds cannot simply be generalized in time and space, they suggest an arthropod bias in the literature.

The abundance of major natural enemies is usually estimated with suction sampling and taken as a measure of the level of biocontrol in rice fields^46^,^47^,^48^,^49^,^50^. The minor role of arthropod predators relative to frogs in our study cannot be attributed to low density, because arthropod predators had a relatively high density at our study site compared to those in unsprayed farmers’ fields in Jiangxi Province (Zou *et al*., unpublished data). We did not find a significant relation between the abundance of arthropod predator taxa in the blower-vac samples and the number of live, mobile BPH removed per taxon. This suggests that care should be taken when interpreting abundance data of taxa of known predators in studies aiming at quantification of field predation levels.

The implications of our study are twofold. Methodologically, the study makes clear that caution must be taken in the interpretation of results from experiments using immobilized sentinels, and provides a strong argument for validation of sentinel methods using video monitoring prior to full-scale application. We also recommend to conduct video monitoring experiments of predation in other major pest-crop systems in which predators are attributed a key role in natural pest suppression but where direct evidence on the identity and relative importance of the predator taxa is still lacking. The second major finding of our study was the important role of frogs in predation of live, mobile BPH. Current strategies to enhance natural suppression of planthoppers in rice focus on arthropod natural enemies (predators as well as parasitoids)^51^,^52^, and a potentially major role of frogs deserves further investigation and scrutiny. Priorities for these follow-up studies concern the effect of BPH prey density at patch and field level and the role of frogs in other major irrigated rice growing areas in Asia.

## Materials and Methods

### Study site and insect material

This study was conducted in two adjacent irrigated rice fields at the Jiangxi Agricultural University (28°46.17′N, 115°49.99′E), Jiangxi Province, China. The first field (400 m^2^) was planted with the hybrid early rice variety PoYou-364 at the end of April 2015, and the second field (300 m^2^) was planted with the hybrid middle rice variety Y-liangyou-1 in mid-June 2015. Both rice varieties are susceptible to BPH and no pesticides were used. Daily precipitation data during the experimental period were obtained from the university’s weather station, located 500 m away from the study fields.

BPH for exposure studies were reared on rice plants in cages (size: 0.4 × 0.65 × 1.5 m) in a greenhouse, using the variety PoYou-364 before July 2015 and Y-liangyou-1 afterwards. The original starting material for the BPH culture was sourced from Nanjing Agricultural University and Yangzhou University.

### Experiment 1

**Figure 4:**
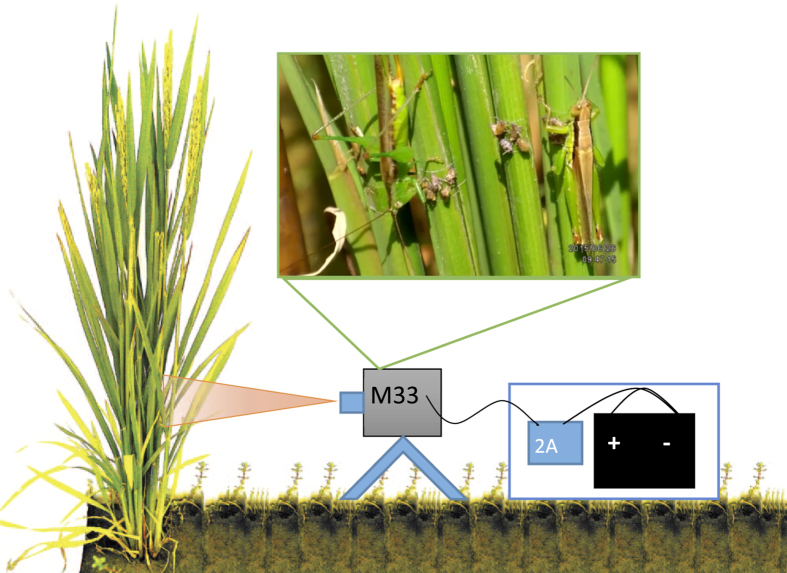
Setup of video monitoring of removal of dead, immobilized brown plant hopper in Experiment 1.

In the first experiment, we used a digital video camera (BenQ^®^, M33) with a resolution of 1920 × 1080 pixels per square inch, a 4.6–46 mm zoom lens and IR night vision function. The camera was powered by a 12 V lead-acid E-bike battery with a capacity of 12 Ah, which allowed at least 45 hours continuous recording (Fig. 4). The camera and battery were connected with a 2 A, 12-to-5V USB-converter and were placed in plastic containers covered with aluminum foil for protection against rain and heat. Data were stored on a 32 G SD card, which was replaced every 8 hours.

Three replicate exposures were done at 48, 55 and 62 days after transplanting. At each time, BPH obtained from the rearing cages were killed by placing them in a freezer at −18 °C for 24 hours. Five 20 cm-long rice stem pieces with a total of 25 adult female BPH (5 per each stem piece) were placed in the field next to a rice plant, at least 3 meters from the edge. BPH were fixed 5 cm from the top of the stem piece, on a 1 cm^2^ double-sided tape.

After placement in the field, the BPH sentinels were monitored over a 72-hour period. A set of BPH sentinels was replaced when fewer than three of the original 25 BPH were left on the stem pieces. In total, over the three replicate exposure trials, we monitored the fate of 250 BPH. The camera was installed at 30 cm distance to obtain the best focus.

### Experiment 2

**Figure 5:**
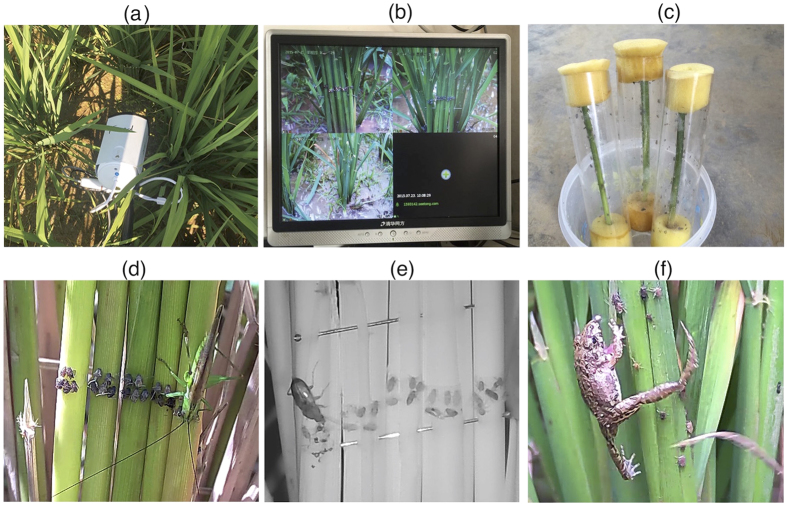
Setup of experiment 2, with camera in the field (**a**); monitoring screen (**b**); transfer tubes with live, mobile brown plant hoppers (BPH) (**c**); long-horned grasshopper feeding on dead, immobilized BPH (**d**); ground beetle feeding on live, immobilized BPH (**e**); and frog catching live, mobile BPH (**f**).

In the second experiment, we used three surveillance cameras (DFD^®^, Shenzhen, China) with a resolution of 1280 × 720 pixels per square inch, an 8 mm fixed lens and IR night vision function (Fig. 5a). The focus distance was set at 30 cm. The cameras were connected to a four-channel Digital Video Recorder (DVR) for data storage, which in turn was connected to a monitoring screen (Fig. 5b). The three cameras were put in the middle of the field, with a spacing of 3 m. We used one camera per treatment.

The three treatments were: (1) dead, immobilized BPH (2) live, immobilized BPH, and (3) live, mobile BPH. In treatment 1, 25 BPH were fixed on rice stem pieces using double-sided sticky tape, as in Experiment 1. In treatment 2, 25 BPH were temporarily (reversibly) immobilized by cooling them at −18 °C for five minutes, and then fixed on rice stems pieces using double-sided sticky tape as in treatment 1. While all BPH were alive when introduced to the field, they eventually died. The time of 50% mortality (excluding predation events) was 3.2 ± 2.4 hours (mean ± SD), and after 16.7 ± 4.7 hours all BPH were dead. For treatment 3, BPH (80% adults and 20% late-instar nymphs with similar body size as adults) were transferred to a tube containing a 20 cm piece of rice stem (Fig. 5c). After 20 minutes, when BPH had settled on the rice stem, the stem pieces were placed in the field, pressed against a rice plant in front of the camera, and the tube was carefully removed. The initial number of BPH in each exposure was 42 ± 18 (mean ± SD). We used a higher initial number of BPH for this treatment than for the immobilized BPH treatments because we anticipated that part of the BPH would move away from the stem pieces after placement in the field. In total, 634 live, mobile BPH were exposed.

After placement in the field, the three treatments were monitored over a 24-hour time period. The above protocol was replicated six times (“observation rounds”), at 32, 47, 59, 67, 80 and 88 days after transplanting, respectively, which covered crop development from tillering to ripening stage. Rice stems with live, mobile BPH were replaced with fresh ones after 8 or 12 hours to compensate for departures and to ensure that sufficient BPH were in the view of the camera to record predation events.

### Monitoring of arthropod abundance

In experiment 1, visual counts of arthropods were conducted on the second day of the video monitoring during five 1-hour periods at the following times: sunrise (04:00–05:00 h), morning (09:00–10:00 h), afternoon (14:00–15:00 h), sunset (19:00–20:00 h), and night (23:00–00:00 h). Night observations were conducted using red light. Counts were performed by two observers, each of them randomly walking through one half of the field where no video recording took place, and recording all encountered predators and other taxa responsible for BPH removal.

In experiment 2, immediately after each observation round, the arthropod community in the field was sampled using a blower-vac (Oleo-Mac BV300, 30.5 cc) that was modified for arthropod sampling^53^,^54^. Each time, six samples were taken using a 40 cm diameter enclosure (0.13 m^2^) and a suction time of two minutes. Due to the relatively small sampling area, we do not expect sampling has influenced the overall arthropod density in the field or affected BPH removal in the observation round.

### Data analysis

The video recordings were reviewed and all BPH removal events were counted. The removal agents were identified at least to family level. We distinguished between “full removal” and “partial removal”. Full removal was defined as the removal of the entire body of a BPH, while partial removal was the removal of only a part of the BPH body. We calculated the success rate for each removal agent as the ratio of the number of successful removal attempts (full and partial) and total number of attempts captured on video, where an attempt was defined as the removal agent being in the view of the camera, from its appearance to its departure, with the removal agent making at least one attempt to capture BPH.

Differences in the number of full removals and partial removals between treatment 1 (dead BPH) and treatment 2 (live immobilized BPH) were analysed by generalized linear mixed models with Poisson error distribution with log-link function^55^, treatment as fixed factor and observation round as random factor. For the overall removals and attempts done by different agents, data from the six observation rounds were pooled and differences between treatments in the success rate of the same taxon were tested with χ^2^ tests. Correlation between the number of a taxon in the blower-vac samples and the number of full removals by this taxon was tested with Spearman’s rank correlation test. Analyses were performed in R^56^, using the “lme4” package^57^.

## Additional Information

**How to cite this article**: Zou, Y. *et al*. Video monitoring of brown planthopper predation in rice shows flaws of sentinel methods. *Sci. Rep.* **7**, 42210; doi: 10.1038/srep42210 (2017).

**Publisher's note:** Springer Nature remains neutral with regard to jurisdictional claims in published maps and institutional affiliations.

## Supplementary Material

Supplementary Information

Supplementary Movie 1

## Figures and Tables

**Table 1:** Overview of a selection of studies using immobilized prey as sentinels.

Prey species	Common name	Prey type	Immobilize method	Study system	Study region	Reference
*Acyrthosiphon pisum*	Pea aphid	Adults	Glued	Barley fields	Sweden	12
*Acyrthosiphon pisum*	Pea aphid	Adults	Glued	Wheat	Eight European countries	13
*Acyrthosiphon pisum*	Pea aphid	Adults	Glued	Several cereal fields	Five European regions	58
*Agrotis ipsilon*	Black cutworm	Larvae	Pinned	Golf courses	Maryland, USA	59
*Agrotis ipsilon*	Black cutworm	Larvae	Pinned	Grassland	Lexington, USA	14
*Cydia pomonella*	Codling moth	Larvae	Tethered	Apple orchard	West Virginia, USA	60
*Diabrotica virgifera*	Corn rootworm	Larvae	Pinned	Maize	South Dakota, USA	61
*Epiphyas postvittana*	Light brown apple moth	Larvae	Pinned	Vineyard	New Zealand	27
*Galleria mellonella*	Wax moth	Larvae	Caged	Garden or fallow fields	Ohio, USA	62
*Heliothis virescens*	Tobacco budworm	Larvae	Pinned	Forest	Arkansas, USA	21
*Plutella xylostella*	Diamondback moth	Larvae	Pinned	Brussels sprout	Netherlands	63
*Spodoptera frugiperda* and *Galleria mellonella*	Fall armyworm and wax moth	Larvae	Pinned or glued	Corn	Michigan, USA	64
*Tenebrio molitor*	Mealworm	Larvae	Glued	Agroforestry system	Indonesia	19
*Trichoplusia ni*	Cabbage looper	Larvae	Glued	Kale	California, USA	65
