# Supplementary material for: Video monitoring of brown planthopper predation in rice shows flaws of sentinel methods
Source: Sci Rep. 2017 Feb 17;7:42210. doi: 10.1038/srep42210 (PMC5314450; doi:10.1038/srep42210)
Supplement: Supplementary Information [file srep42210-s1.docx]

**Video monitoring of brown planthopper predation in rice shows flaws of sentinel methods**

*Running title: Video monitoring of predation*

Yi Zou^1,2^, Joop de Kraker^3^, Felix J. J. A. Bianchi^4^, Mario D. van Telgen^1^, Haijun Xiao^5,†^, Wopke van der Werf^1^

1. Centre for Crop Systems Analysis, Wageningen University, Wageningen, The Netherlands
2. Current address: Department of Environmental Science, Xi’an Jiaotong-Liverpool University, Suzhou, China
3. Department of Science, Open University, Heerlen, The Netherlands
4. Farming Systems Ecology, Wageningen University, Wageningen, The Netherlands
5. Institute of Entomology, Jiangxi Agricultural University, Nanchang, China

† Author for correspondence:

Haijun Xiao, hjxiao@jxau.edu.cn

**Supplementary materials**

Supplementary material 1. Experiment 1: total number of individuals per taxon counted in visual assessment of arthropod abundance (relative density), number of full removals and partial removals (between brackets the number of successful attempts) and success rates for full removals; NA: not applicable.

| **Removal causes** | **Relative density** | **Full removals** | **Partial removals** | **Success rate** |
| --- | --- | --- | --- | --- |
| Acrididae | 201 | 5(1) | 0 | 1.00 |
| Carabidae | 14 | 2(1) | 1(1) | 1.00 |
| Linyphiidae | 389 | 0 | 5(5) | 0.16 |
| Lycosidae | 204 | 13(10) | 5(5) | 0.93 |
| Salticidae | 58 | 0(0) | 3(3) | 0.60 |
| Sciomyzidae | 262 | 5(4) | 14(14) | 0.15 |
| Staphylinidae | 389 | 0 | 2(2) | 1.00 |
| Tettigoniidae | 490 | 142(20) | 11(11) | 0.95 |
| Succineidae | NA | 0 | 1(1) | 1.00 |
| Rain | NA | 1(1) | 0 | 1.00 |

Supplementary material 2. Experiment 2: total number (relative density) of individuals per arthropod taxon in blower-vac suction samples (n=36), number of full removals and partial removals (between brackets the number of successful attempts) and success rates for full removals, for three treatments (DI: dead, immobilized; LI: live, immobilized; LM: live, mobile); NA: not applicable.

| **Removal causes** | **Relative density** | **Treatment** | **Full removals** | **Partial removals** | **Success rate** |
| --- | --- | --- | --- | --- | --- |
|  |  | DI | 9(7) | 19(19) | 0.84 |
| Carabidae | 38 | LI | 54(16) | 15(15) | 0.65 |
|  |  | LM | 7(5) | 0 | 0.24 |
|  |  | DI | 0 | 0 | NA |
| Formicidae | 4 | LI | 0 | 0 | 0 |
|  |  | LM | 0 | 0 | NA |
|  |  | DI | 0 | 1(1) | 1 |
| Gryllidae | 0 | LI | 0 | 0 | NA |
|  |  | LM | 0 | 0 | NA |
|  |  | DI | 0 | 0 | NA |
| Linyphiidae | 30 | LI | 0 | 0 | 0 |
|  |  | LM | 1(1) | 0 | 1 |
|  |  | DI | 0 | 0 | NA |
| Locustellidae | NA | LI | 0 | 0 | NA |
|  |  | LM | 8(2) | 0 | 1 |
|  |  | DI | 0 | 1(1) | 0.5 |
| Lycosidae | 31 | LI | 5(1) | 3(3) | 1 |
|  |  | LM | 2(2) | 0 | 1 |
|  |  | DI | 0 | 0 | 0 |
| Ranidae | NA | LI | 0 | 0 | 0 |
|  |  | LM | 71(40) | 0 | 0.93 |
|  |  | DI | 0 | 0 | 0 |
| Salticidae | 10 | LI | 1(1) | 3(3) | 0.8 |
|  |  | LM | 1(1) | 0 | 1 |
|  |  | DI | 2(2) | 6(6) | 0.21 |
| Sciomyzidae | 3 | LI | 0 | 4(4) | 0.27 |
|  |  | LM | 0 | 0 | NA |
|  |  | DI | 0 | 0 | 0 |
| Staphylinidae | 2 | LI | 0 | 0 | 0 |
|  |  | LM | 2(2) | 0 | 0.5 |
|  |  | DI | 20(2) | 1(1) | 1 |
| Tettigoniidae | 1 | LI | 0 | 0 | NA |
|  |  | LM | 0 | 0 | NA |
|  |  | DI | 0 | 0 | NA |
| Escape | NA | LI | 3(3) | 0 | 1 |
|  |  | LM | 0 | 0 | NA |
|  |  | DI | 13(3) | 0 | 1 |
| Rain | NA | LI | 3(2) | 0 | 1 |
|  |  | LM | 1(1) | 0 | 1 |
